# Crossed-reflex in antiphospholipid chorea

**DOI:** 10.1007/s10072-024-07622-5

**Published:** 2024-06-19

**Authors:** Joan Miquel Fernández-Vidal, Gonzalo Olmedo-Saura, Luis Antonio Querol-Gutiérrez, Jaime Kulisevsky, Jesús Pérez-Pérez

**Affiliations:** 1 Department of Neurology, Hospital de La Santa Creu I Sant Pau, Barcelona, Spain; 2 Neuromuscular Diseases Unit, Department of Neurology, Hospital de La Santa Creu I Sant Pau, Barcelona, Spain; 3 Network Research Center-Rare Diseases (CIBERER), Madrid, Spain; 4 Movement Disorders Unit, Department of Neurology, Hospital de La Santa Creu I Sant Pau, 90 Mas Casanovas St 08040, Barcelona, Spain; 5 Network Research Center, Neurodegenerative Diseases (CIBERNED), Madrid, Spain; 6 Department of Medicine, Barcelona Autonomous University, Barcelona, Spain

**Keywords:** Chorea, Antiphospholipid syndrome, Crossed-reflex

## Abstract

Chorea is a hyperkinetic movement disorder associated with various underlyingconditions, including autoimmune diseases such as antiphospholipid syndrome (APS). APS can manifest with a wide range of neurological symptoms, including chorea. We present a case of a 77-year-old man with subacute generalized chorea secondary to primary APS. Notably, the patient exhibited a left patellar crossed-reflex, a phenomenon rarely documented in chorea cases, the pathophysiology of which has not yet been elucidated. In summary, this case challenges the traditional demographics of antiphospholipid syndrome (APS) by suggesting a potential link between APS and late-age patients. It emphasizes the importance of considering APS in late-onset chorea cases.

**Supplementary Information:**

The online version contains supplementary material available at 10.1007/s10072-024-07622-5.

Chorea is a hyperkinetic movement disorder that can be caused from diverse conditions including autoimmune diseases such as antiphospholipid syndrome (APS) and also has been associated with the isolated presence of antiphospholipid antibodies (aPL) [1, 2].

APS can manifest with neurological symptoms like cerebrovascular accidents, thrombosis, seizures, and movement disorders. Although chorea's prevalence in APS is approximately 1.3%, its pathophysiological mechanisms remain unclear [1]. Postulated mechanisms include autoantibody-induced endothelial dysfunction, inflammation, and microthrombosis, or immune-mediated attack against basal ganglia epitopes [2, 3].

The presentation of chorea is usually subacute with a monophasic course and choreic movements can be focal, unilateral or generalized [3].

We present the case of a 77-year-old man with a history of atrial fibrillation anticoagulated with apixaban and severe mitral insufficiency treated with annuloplasty; who was admitted due to subacute generalized chorea (video 1, segment 1). After pharmacological control of the chorea with tetrabenazine, neurologic examination revealed the presence of a left patellar crossed-reflex that was elicited when exploring deep muscle reflexes in both the upper limbs and the contralateral half of the body (video 1, segment 2). Rest of the neurological examination was within normal limits.

Laboratory investigations revealed high serum anti B2 glycoprotein-I IgG titers (Table 1). No spinal cord or basal ganglia lesions were found in the magnetic resonance imaging (MRI, video 2), but they did find them at an extra-striatal level including periventricular white matter, right cerebellum, left thalamus and both semioval centers. HTT gene sequencing showed a CAG repeats in a normal range. Surface electromyography showed a monophasic contraction of left quadriceps (video 1, segment 3).

**Table 1:** Analysis and laboratory tests conducted during admission for possible causes of chorea

Test	Result	Normal Range
Sodium (Na)	136 mmol/L	136—145 mmol/L
Calcium (Ca)	2.43 mmol/L	2.20—2.50 mmol/L
Urea	7.8 mmol/L	3.0—9.2 mmol/L
Thyrotropin	1.16 mUI/L	0.30—5.00 mUI/L
Glycated Hemoglobin (HbA1c)	5.5%	4.6—5.8%
Antinuclear and cytoplasmic antibodies (ANA)	1:160	
Antineutrophil cytoplasmic antibodies (ANCA)	Negative	
Anti-cardiolipin antibody IgG	5.60 U/mL	0.00—10.30 U/mL
Anti-cardiolipin antibody IgM	4.70 U/mL	0.00—26.00 U/mL
Anti-β2 glycoprotein-I IgG	**39.10 U/mL**	0.00—13.20 U/mL
Anti-β2 glycoprotein-I IgM	1.10 U/mL	0.00—26.99 U/mL
Antistreptolysin O (ASLO)	Negative	
Lupus anticoagulant (Silica clotting time)	Negative	
Lupus anticoagulant (Russell's viper venom time)	Negative	
HTT gene—Huntington	Negative (18 CAG trinucleotide repetitions)	
Anti-neuronal surface antibodies (NMDA, AMPA, GABAa, GABAb, mGluR1, mGluR5, DPPX, IgLON5, Neurexin, LGI1 and CASPR2)	Negative	

After etiological study, including a comprehensive neurovascular study with no other relevant findings, the patient was diagnosed as chorea secondary to primary antiphospholipid syndrome.

Tetrabenazine was started at ascending doses until tetrabenazine 12.5 mg thrice a day. The patient showed a marked improvement and at the 3 and 6-month follow-up visits, he only presented mild appendicular chorea of the left leg when performing activation maneuvers. At the one-year follow-up visit, the patient exhibited no choreic movements, and the dosage of tetrabenazine was progressively reduced until it was discontinued. He also maintains control with Hematology and in the control analyses there is a persistent increase in the titers of anti B2 glycoprotein-I IgG and the patient is undergoing anticoagulant treatment.

## Discussion

Despite the fact that APS is more frequent in young female patients [1], this case in a late-age patient suggests the possible relationship with a second peak of autoimmune diseases in the context of senescence [4] which recommends the screening for APS in late-onset chorea.

Crossed-reflexes pathways in lower extremities were described more than a century ago in mice but there is no clear pathophysiologic explanation, especially for those crossed up-down limb, in humans [5]. Crossed patellar reflex with or without a polyphasic contraction pattern (hung up reflex) can be presented in other types of chorea as Huntington’s disease [6] and its pathophysiology have not been elucidated yet. Revising published literature, we did not find cases in antiphospholipid chorea.

## Conclusion

This case challenges conventional assumptions about antiphospholipid syndrome (APS) by highlighting its potential association with late-age patients presenting with chorea. It underscores the significance of considering APS in the differential diagnosis of late-onset chorea cases. Furthermore, the intriguing presence of crossed-reflexes, exemplified by the left patellar crossed-reflex in this case, raises questions about their pathophysiology in chorea, an area that warrants further investigation.

### Supplementary Information

Below is the link to the electronic supplementary material.

Supplementary file1 (MP4 9.78 MB)
